# COVID-19 airborne transmission: a new frontier of infection

**DOI:** 10.3126/nje.v11i3.40000

**Published:** 2021-09-30

**Authors:** Indrajit Banerjee, Jared Robinson

**Affiliations:** 1, 2 Sir Seewoosagur Ramgoolam Medical College, Belle Rive, Mauritius

##  

Sir,

The SARS-CoV-2 virus which has spread globally and now claimed the lives of 4.5 million people, may be mutating to become more virulent and thus should be a growing concern for both policy makers and individuals alike. There is compelling evidence which suggests that the newer variants of the virus may be spreading more via fine aerosol particles as compared to their predecessors [1].

### Viral transmission

The mechanism of transmission of the virus has been a topic of intense debate among physicians, as previously it was understood that the initial variants of the virus were generally isolated to heavier droplets which would sink and settle on a surface fairly rapidly and thus be spread directly through contact, other routes of transmission via the faeco-oral route have also been established. Aerosol transmission of the virus has remained a somewhat of a grey area; however new-found research and studies conducted on the newer variants of the SARS-CoV-2 virus indicate that the aerosol route of transmission is being utilized [2].

### Mechanism of aerosol production

Aerosols can be produced from various bodily excreta and secretions which contain the SARS-CoV-2 virus. These secretions and excreta which contain the virus can be aerosolized into infectious particles in a multitude of mechanisms. Various secretions chiefly those such as respiratory secretions are aerosolized via menial everyday tasks such as sneezing, talking, exhaling and coughing. Various surgical and interventional procedures such as non-invasive ventilation, tracheal intubation and bronchoscopies also provide the necessary mechanical energy to form aerosolized particles and thus increase the risk to medical personnel performing such procedures.[3] Superadded to this, the deposits of previously aerosolized material can be re-aerosolized by everyday actions such as walking and door opening. Laboratory contamination can occur from biological specimens which have become aerosolized due to failure of the adherence of proper safety protocol and procedures [4,5].

### Aerosol transmission

Studies conducted at the University of Maryland have concluded that individuals who were infected with the alpha variant of the virus carried up to eighteen times the amount of viral RNA in the aerosols they produced as compared to the older and less virulent variants. The load of viral RNA in these aerosols being high enough to culture the virus from the air samples taken in the laboratory. This evidently relating to a more virulent strain, depicting how the virus has mutated to increase its repertoire of transmission [6].

Supplementary evidence from studies undertaken in Singapore which compared fine (≤5μm) and coarse (>5μm) respiratory aerosols produced when breathing, talking, and singing concluded that the fine aerosols contained a greater amount of RNA viral copies than the coarse aerosols. The importance of this finding is rooted in pathophysiology; as particles less than 6μm in size are able to overcome the respiratory systems natural defences and thus can reach the deeper segments and lobes of the lungs. An alarming factor is that the buoyancy of these fine aerosols means that the virus can remain airborne for a prolonged period after being aerosolized and thus adds a new dimension to the risk of infection in poorly ventilated spaces. Due to the buoyancy of these fine aerosolized particles, the capability of them to remain airborne for extended periods increases the likelihood of inoculation into a desired host (i.e., man). These fine aerosolised particles therefore drastically increase the risk to those individuals not wearing masks [7].

Greenhalgh T et. al, has published 10 scientific reasons in the support of the airborne transmission of COVID-19. 1) Superspreading events account for a substantial number of COVID-19 infections. On analysis of human behaviour in such events it has been concluded that long-range transmission of the virus has taken place in settings where the production of aerosolized particles is maximal, thus supporting the notion of airborne transmission. 2) The long range transmission of the virus to people in separate rooms in quarantine hotels has been recorded. 3) Asymptomatic and pre-symptomatic transmission account for roughly one third of the cases globally where individuals spread the virus through speaking, singing etc. 4) Transmission of the virus is higher indoors than outdoors. 5) Nosocomial infections have been recorded where strict protocol and use of PPE for droplet, but not aerosolized infection has been adhered to. 6) Viable SARS-CoV-2 has been isolated in air samples from bedrooms occupied by COVID-19 patients, where no invasive or particulate generating procedure has been performed.7) The SARS-CoV-2 virus has been detected in building ducts and hospital filters which can only be reached via aerosolized particulate matter. 8) Studies involving infected animals, with interconnecting ducts systems to cages with healthy animals have shown the transmission of the virus which can thus only be logically explained via aerosols. 9) No study has provided strong evidence to consistently refute aerosol transmission. 10) Limited evidence is available to support other predominant routes of infection. The assumption that transmission through close physical contact and proximity is flawed. Large respiratory droplets or fomites have historically (for decades) been used to deny the airborne transmission of tuberculosis and measles which has subsequently been proven otherwise [8].

As of May 18, 2021 the WHO officially updated and recognized aerosol transmission as one of the routes used by the virus to infects its host, this was instituted after over 200 experts wrote an open letter to the WHO insisting that the evidence of airborne transmission of the virus particularly through aerosols had to be recognized [9].

## Conclusion

The newly found evidence and acceptance of fine aerosol transmission in the newer variants of the SARS-CoV-2 virus should be a growing concern for both policy makers and individuals alike. By virtue of the fact that the virus spreads through such means, increases the risk of transmission and contamination highly as the virus can be transmitted via long range and through common ventilation and duct systems. In light of this it is now pertinent for legislation to support the use of personal protection equipment to better safeguard the health of the public.

Indrajit Banerjee, Sir Seewoosagur Ramgoolam Medical College, Belle Rive, Mauritius

Jared Robinson, Sir Seewoosagur Ramgoolam Medical College, Belle Rive, Mauritius

Dated the 20 September 2021
